# Cycle Threshold Values as Indication of Increasing SARS-CoV-2 New Variants, England, 2020–2022

**DOI:** 10.3201/eid2910.230030

**Published:** 2023-10

**Authors:** Rebecca E. Harrison, Ahmed Hamada, Nujcharee Haswell, Aigul Groves, Karina-Doris Vihta, Kerry Cella, Sarah Garner, Ann Sarah Walker, Anna C. Seale

**Affiliations:** United Kingdom Health Security Agency, London, UK (R.E. Harrison, A. Hamada, N. Haswell, A. Groves, K. Cella, S. Garner, A.C. Seale);; University of Oxford, Oxford, UK (K.-D. Vihta, A.S. Walker);; Warwick Medical School, University of Warwick, Coventry, UK (A.C. Seale);; London School of Hygiene & Tropical Medicine, London (A.C. Seale)

**Keywords:** COVID-19, 2019 novel coronavirus disease, coronavirus disease, severe acute respiratory syndrome coronavirus 2, SARS-CoV-2, viruses, respiratory infections, polymerase chain reaction, quantitative real-time PCR, zoonoses, England, United Kingdom

## Abstract

Early detection of increased infections or new variants of SARS-CoV-2 is critical for public health response. To determine whether cycle threshold (Ct) data from PCR tests for SARS-CoV-2 could serve as an early indicator of epidemic growth, we analyzed daily mean Ct values in England, UK, by gene target and used iterative sequential regression to detect break points in mean Ct values (and positive test counts). To monitor the epidemic in England, we continued those analyses in real time. During September 2020–January 2022, a total of 7,611,153 positive SARS-CoV-2 PCR test results with Ct data were reported. Spike (S) gene target (S+/S−)–specific mean Ct values decreased 6–29 days before positive test counts increased, and S-gene Ct values provided early indication of increasing new variants (Delta and Omicron). Our approach was beneficial in the context of the first waves of the COVID-19 pandemic and can be used to support future infectious disease monitoring.

From identification of SARS-CoV-2 in December 2019 through September 22, 2022, ≈612 million confirmed cases and 6.5 million confirmed deaths were reported worldwide (*1*). During the COVID-19 pandemic, epidemic waves have usually been associated with emergence of a new variant. Assessing the emergence of any such new variant is critical for public health response. Although testing is essential for monitoring trends in detected cases, the diagnostic real-time quantitative reverse transcription PCR (qRT-PCR) tests also provide data pertaining to viral load and presence or absence of particular genes in the virus detected, which may further aid assessment of the likely course of the epidemic.

qRT-PCR results are sensitive, based on detection of SARS-CoV-2 RNA. Results are positive, negative, or indeterminate, based on a threshold for the number of replication cycles required for detection, the cycle threshold (Ct) (*2*,*3*). Typically, the higher the Ct value, the lower the viral load in the specimen (*4*).

Viral load is associated with infectivity and can be associated with severity of illness (*5*–*7*). Low Ct values have been associated with testing just after symptom onset (*8*–*11*), having the classic symptoms (e.g., cough/fever/anosmia/ageusia) (*4*), increased duration of viral shedding, (*12*,*13*), severe case of COVID-19 and increased risk for critical illness and death (*14*–*19*), older age (*20*–*22*), vaccination status (*23*), and higher secondary attack rate (*7*,*24*).

Individually, interpretation of Ct values as a proxy of infectiousness or severity of illness should be approached with caution. Results may be influenced by the time course of infection, additional technical factors during sampling, types of processing, and the specific assay used (*25*,*26*). However, in the population, Ct values as a proxy for viral load may provide information on the growth of the epidemic, if measured with a standardized assay.

On March 27, 2020, a new network of Lighthouse laboratories, was set up for SARS-CoV-2 testing in the United Kingdom (J.A. Douthwaite, unpub. data, https://www.researchsquare.com/article/rs-637020/v1). Four of those laboratories used the Thermo Fisher TaqPath RT-PCR test (*27*), which detects 3 SARS-CoV-2 gene targets: open reading frame (ORF)1, nucleocapsid (N), and spike (S). Ct values are available for each gene detected, and RT-PCR tests at TaqPath are standardized and thus comparable across laboratories. All 3 gene targets are not always detected, which can result from a sample of low quality or low viral load or from a virus having mutations in the ORF1ab, S, or N gene.

Over the course of the epidemic, and in particular with the emergence of the Alpha variant, which had S gene target failure (SGTF/S−) compared with the initial wild-type variant, the potential use of the S gene for identifying emergence of a new variant, combined with analysis of Ct values to investigate growth, became apparent. Data from both National Health Service (NHS) Test and Trace and from the UK COVID-19 Infection Survey (CIS) were thus assessed for the presence or absence of the S gene (*4*,*28*) (A.S. Walker, unpub. data, http://medrxiv.org/lookup/doi/10.1101/2021.01.13.21249721).

We describe use of Ct values with S gene target data for early detection and community growth of the Alpha, Delta, and Omicron (BA.1) SARS-CoV-2 variants in England; that approach was used in real-time monitoring for Delta and Omicron. We conducted our analysis to provide information for the outbreak response to the COVID-19 pandemic. Work was undertaken in accordance with national data regulations. We accessed and used only fully anonymized data from the UK Health Security Agency (UKHSA) in a secure research environment.

## Methods

### Data Source

We used data from the England national clinical and community testing program for September 1, 2020, through January 31, 2022 (*29*). We monitored SARS-CoV-2 RT-PCR tests processed at Taqpath laboratories at Milton Keynes, Glasgow Central, Alderley Park, and Newcastle. Samples were reported as positive if the algorithm was interpreted as positive for >1 of the N or ORF1ab genes by an assay-specific algorithm and decision mechanism that analyzes the raw assay data. The S gene was not considered a prerequisite for positivity because of mutations detected since mid-May 2020. TaqPath laboratories almost exclusively use community-based tests rather than healthcare-based tests.

### Data Analyses

As part of routine monitoring, we prepared the data in SQL and conducted analyses in R (The R Project for Statistical Computing, https://www.r-project.org) and Python (https://www.python.org) during February 2020–February 2022 and present analyses through February 2022. We describe the total positive SARS-CoV-2 PCR tests, the proportion processed in Taqpath laboratories, and the changes in mean Ct values with different gene target combinations.

The absence of the S gene (S−) was a proxy for Alpha (September 1, 2020–January 28, 2021) and Omicron BA.1 (October 1, 2021–January 31, 2022), whereas the presence of the S gene (S+) with both other genes was a proxy for Delta (February 1, 2021–January 5, 2022). To confirm the use of the S gene as a proxy, we linked those data to whole-genome sequencing and rapid genotyping data, if available. Rapid genotyping was conducted 3–5 days after PCR and whole-genome sequencing data 10–14 days after PCR.

We recorded absolute numbers of positive test results and proportions by variant and measured 7-day rolling means of positive test results by variant by calendar period during which variants were detected and started to grow: Alpha (September 1, 2020–January 31, 2021), Delta (February 1, 2021–November 30, 2021), and Omicron (October 1, 2021–January 31, 2022). We restricted analysis of counts of S− and S+ to samples with Ct <30 because absence of S-gene detection above that threshold could reflect stochastic variation at low viral loads. Mean daily Ct values included all positive samples, including those with Ct >30, which enabled better detection of drops from high Ct values.

To detect trends and break points in Ct values and numbers of positive test results, we applied iterative sequential regression (ISR), which allows for changes in trend to be identified in real time through its sequential design (*28*), to daily mean Ct values and daily numbers of positive test results for each calendar period, broken down by S gene target profile (ORF1ab, N, and S+ vs. ORF1ab, N, and S−). If a model with 2 trends was a better fit compared with 1 trend, we fixed the change point and repeated the process (i.e., added more data and new models with this change point, as well as other potential change points after the initial one was fitted). That method enables efficient estimation of multiple changes in trend, unlike a traditional grid search algorithm. We set break points to be at least 14 days apart and used a gamma model because it seemed to be the most appropriate on the basis of its visual fit, despite the apparent dispersion constraints, and the negative binomial or Poisson may also be appropriate for the number of positive test results. We used results from the ISR model to describe days passed between break points in mean Ct trend and daily numbers of positive test results and the time taken for the break point to be identified by the ISR model.

## Results

### Emergence of Alpha from Wild-Type Variants

During April 2020–March 2021, a total of 3,312,159 PCR tests for SARS-CoV-2 were conducted in England through clinical and community testing streams; 27,902 (0.84%, 95% CI 0.83%–0.85%) tests were positive. Over that period, the percentage of swab samples with no S gene detected increased; >86% of those with Ct <30 had no S gene detected from November 16, 2020, through March 31, 2021, concurrent with emergence and expansion of Alpha (B.1.1.7) (although less complete dominance compared with later variants) (*4*). That finding led to use of the S gene Ct values, case numbers, and proportions as a proxy for monitoring positive tests that did or did not detect an S gene, according to both the UK Office of National Statistics Covid Infection Survey (ONS CIS) and UKHSA surveillance data (*4*,*28*). The S− characteristic was later also found to be a characteristic of Omicron BA.1. Other variants, such as Delta, Beta, and Gamma, usually do not have mutations in region of the S gene detected by the TaqPath assay primers, and so the PCR test will usually detect the S, ORF1ab, and N genes, described as S+.

### Emergence of New Variants in Terms of S Gene and Growth (Ct Values)

**Figure 1:**
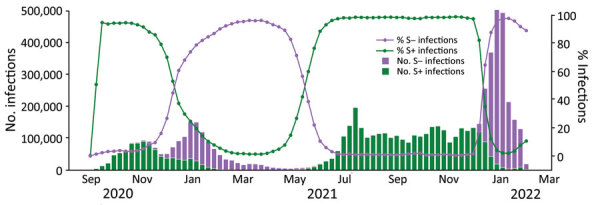
Weekly Taqpath reverse transcription quantitative PCR (*27*) gene detection (S+/S−) in SARS-CoV-2 infections, England, August 31, 2020–January 31, 2022. The chart excludes cycle threshold (Ct) values >30 for S− because when Ct >30, the S− may be caused by a sample of low quality or with low viral load rather than a reliable S− signal. Test results for which only 1 gene is detected are excluded. S, spike; S+, presence of S gene; S−, absence of S gene.

During September 1, 2020–January 31, 2022, a total of 15,139,217 positive RT-PCR test results were reported in England, of which 7,611,153 (50.3%) had Ct values available for ORF1ab, N, or S gene targets from a TaqPath RT-PCR assay. Infections with S– virus were dominant from November 2020 through April 2021 (Alpha), and S+ infections became dominant after April 2021 (Delta), and then S− infections were dominant again between from 2021 through January 2022 (Omicron BA.1) (Figure 1). From January on, the BA.2 (S+) variant started to emerge. The sequence data and rapid genotyping results, when later available, were closely aligned (Appendix Figure).

**Figure 2:**
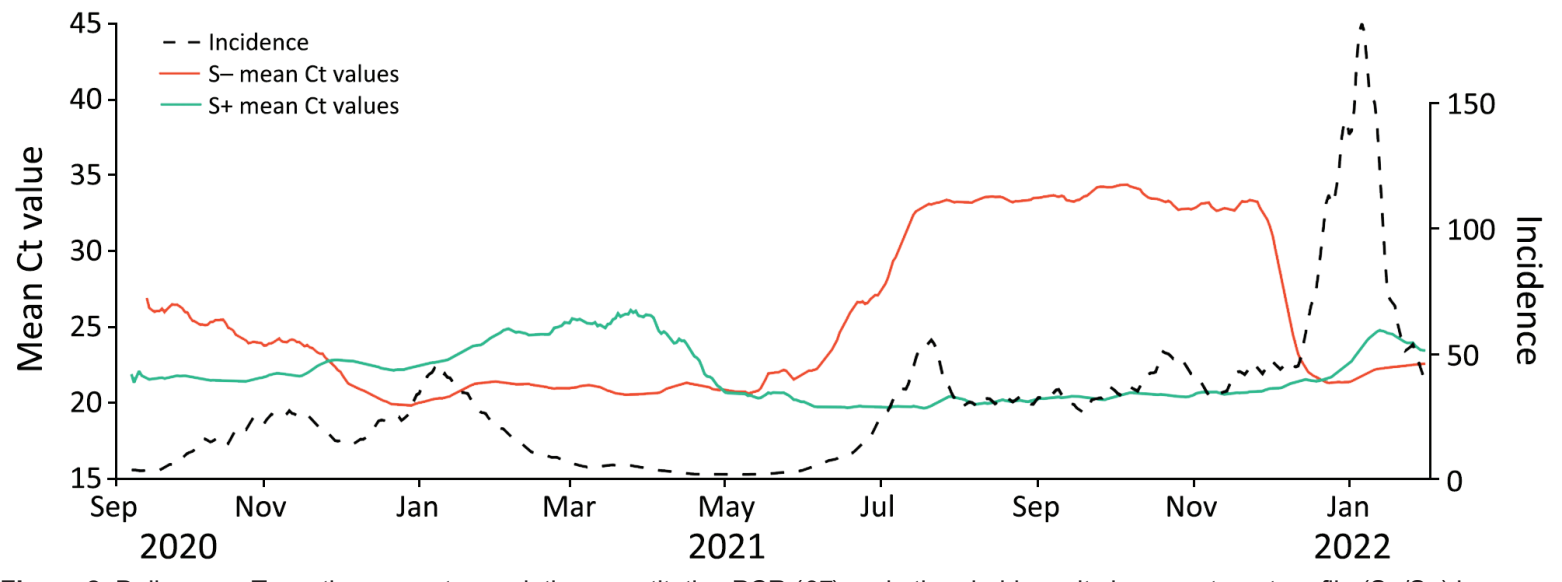
Daily mean Taqpath reverse transcription quantitative PCR (*27*) cycle threshold results by gene target profile (S+/S−) in SARS-CoV-2 infections, England, August 31, 2020–January 31, 2022. S+ indicates open reading frame ab1, nucleocapsid, and S detected; S− indicates open reading frame ab1 and nucleocapsid detected. Incidence, cases/100,000 population; S, spike; S+, presence of S gene; S−, absence of S gene.

Ct values in S+ tests slowly increased from September 2020 through March 2021 as wild-type SARS CoV-2 incidence decreased, and mean Ct values in S− tests decreased from September through December 2020 as Alpha increased. Ct values in S+ tests decreased as Delta incidence increased from May 2021. In December 2021, mean Ct values for S− tests again decreased rapidly, just as Omicron BA.1 incidence increased. (Figure 2). 

### Emergence of Alpha 

From September 1, 2020, through January 31, 2021, daily S− test results (proxy Alpha) rose from 1 to 6,487, peaking at 22,936 on January 2, 2021. Sequencing of 169,823 (4.7%) of 3,617,137 viruses over this period showed that 78,410 (46.2%) were Alpha (Appendix Table 1). With the growth of Alpha, the daily mean Ct value for S− tests decreased from 26.1 on September 1, 2020, to 19.6 on December 20, 2020.

**Figure 3:**
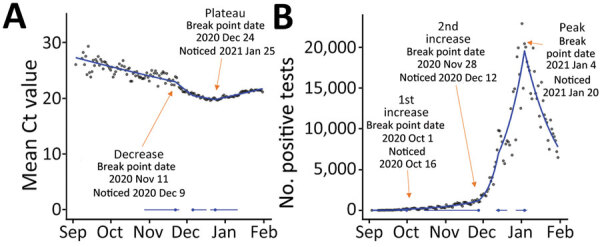
Emergence of novel Alpha variant of SARS-CoV-2 in England, showing mean Ct values (A) and positive results for SARS-CoV-2 S tests (B) for September 3, 2020–January 31, 2021, according to a gamma model. Break points detected through iterative sequential regression (ISR) that indicate significant changes in mean Ct values and positive test counts are labeled. Blue line represents estimated S− mean Ct value and positive test counts by ISR. Blue lines at the base of the graph represent 95% CIs around the break points estimated by the ISR model. Ct, cycle threshold; S, spike; S+, presence of S gene; S−, absence of S gene.

At that time, we were not using ISR in real time, but as we implemented the approach in mid-2021, we detected the first break point in mean daily Ct values in S− tests on November 22, 2020 (and it took 14 days for the ISR model to notice this break point), which was ≈6 days before incidence increased further. The drop in Ct value, if observed in real time, could have provided an early indicator of the Alpha increase. Retrospectively, we detected a break point for the plateau/increase in Ct values on December 24, 2020 (noticed 32 days later), and the peak in positive tests 11 days later on January 4, 2021 (noticed 16 days later) (Figure 3).

### Emergence of Delta 

From February 1, 2021, through November 30, 2021, daily S+ tests increased from 544, to 21,797, peaking at 35,866 on July 15, 2021. Sequencing of 1,546,168 (25.9%) of 5,965,964 viruses over that period showed that 1,273,965 (82.4%) were Delta (Appendix Table 1). 

**Figure 4:**
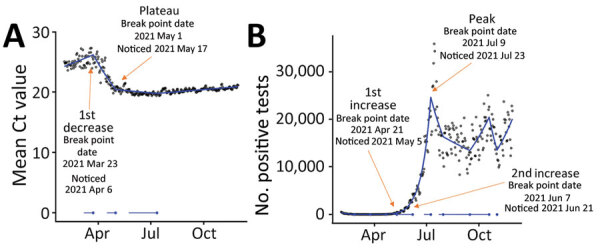
Emergence of novel Delta variant of SARS CoV-2 in England, showing mean Ct values (A) and positive test counts for SARS CoV-2 S-gene–positive tests (B) for February 1, 2020, through November 30, 2021, according to a gamma model. Break points detected through iterative sequential regression (ISR) that indicate significant changes in mean Ct values and positive tests are labeled. Blue line represents estimated S− mean Ct value and positive test counts by ISR. Blue lines at the base of the graph represent 95% CIs around the breakpoints estimated by the ISR model. Ct, cycle threshold; S, spike; S+, presence of S gene; S−, absence of S gene.

During this period, through regular analysis in real time, we first detected a break point in mean Ct values in S+ tests on March 23, 2021 (noticed 14 days later), ≈29 days before incidence started increasing. We detected a break point for the plateau/increase in Ct values on May 1, 2021 (noticed 16 days later), and the peak in positive test results 69 days later on July 9, 2021 (noticed 14 days later) (Figure 4). Use of gene target–specific data was particularly valuable for early detection of Delta growth at that time because overall case counts were declining in the country, masking growth of the new variant. The Beta and Gamma variants also emerged globally around this time, and our analyses provided early indication that these variants were not growing rapidly in England.

### Emergence of Omicron 

During October 1, 2021−January 31, 2022, the daily number of S− tests increased from 10 to 17,237, peaking at 108,438 on January 4, 2022. Sequencing of 1,961,850 (25.1%) of 7,800,924 viruses over that period showed that 1,120,807 (57.1%) were Omicron BA.1 (Appendix Table 1).

**Figure 5:**
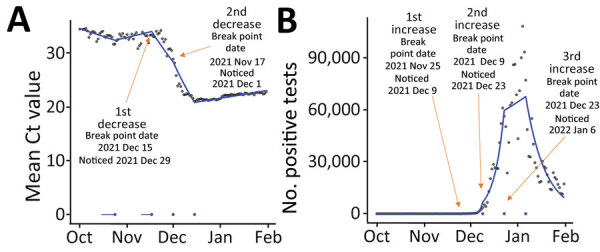
Emergence of novel Omicron (BA.1) variant of SARS CoV-2 in England, showing mean Ct values (A) and positive test counts for SARS CoV-2 S–tests (B) from October 1, 2021, through January 31, 2022, according to a gamma model. Break points detected through iterative sequential regression (ISR) that indicate significant changes in mean Ct values and positive tests are labeled. Blue line represents estimated S− mean Ct value and positive test counts by ISR. Blue lines at the base of the graph represent 95% CIs around the break points estimated by the ISR model. Ct, cycle threshold; S, spike; S+, presence of S gene; S−, absence of S gene.

During that period, through regular analyses in real time, we first detected a break point in mean Ct values in S− tests on November 17, 2021 (noticed 14 days later), ≈8 days before incidence started increasing. We again observed a drop in Ct value as a precursor to the start of the increasing number of positive test results. Over that period, mean Ct values for S− tests decreased from 34.5 to 22.9 from October 1, 2021, through January 31, 2022, dropping to a low of 21.0 on December 15, 2021 (Figure 5).

For S+ tests over the same period, we observed mean Ct values increase from 20.6 to 23.2 and daily positive tests decrease from 13,597 to 353. We also observed Ct values starting to drop again toward the middle of January 2022, resulting from emergence of Omicron BA.2.

## Discussion 

Detecting emergence and growth of new SARS CoV-2 variants is valuable for public health response. Starting in February 2022 in England, knowing the patterns for Alpha, we implemented routine monitoring of positive SARS-CoV-2 clinical test results in the community, by S gene status and mean Ct values, which subsequently provided an early indication of increased incidence for Delta and Omicron. We observed decreasing mean gene target –Ct values in surveillance data ≈6–29 days before variant-specific incidence increased in the population. Mean Ct values plateaued or slightly increased ≈1 month before peak case incidence.

Although the changes in Ct values and S gene data were useful early indicators of increasing incidence, they could not be interpreted in isolation. We needed confirmation of the use of the S gene as a proxy marker from genotyping and whole-genome sequencing. It was also fortuitous that for each new variant that became dominant in the United Kingdom during 2020–2021, its presence alternated with absence of the S gene. Tracking the growth of Delta in the United Kingdom by using S gene data was straightforward because no other S+ cases were in wide circulation at the time. A study in the United States, where Beta had gained traction along with Delta, was not able to use S gene data in the same way that was possible here (*30*). The S gene is not, however, necessarily a prerequisite for using changes in Ct values to monitor emerging variants; although detection times may be slower, changes in Ct value trends should be detectable in positive tests, unrestricted by gene target (*31*). However, in the period that this work was undertaken (up to and including Omicron BA.1), we did not observe and thus analyze epidemic changes in which the S gene target did not change, and thus we cannot compare the time taken to detect changes by using this method without a change in gene target.

The sample size needed is also relevant to the technique. Although we do not present the results here, we were able to use ISR for the 9 regions of England to detect and compare rates of early growth in new variants. However, when this technique was attempted for all 309 lower tier local authorities, absolute and relative weekly S+/S− case numbers were usually more useful than Ct values for tracking growth because of the low number of positive test results during the emergence of a new variant at this geographic level. Going forward, to determine how quickly changes could be detected on a smaller scale, testing this approach on smaller datasets may be useful. Pandemic monitoring benefited by laboratories using a specific, standardized assay, although coverage varied regionally; coverage was notably lower in southwestern England. The use of the standardized TaqPath assay reduced data variation, which may be introduced if different assays or thresholds are used. A study in Bahrain used results from a variety of different assays and found utility of Ct values for predicting the pandemic trajectory to be lower than we did, although that finding may also be associated with their application of a different modeling technique (*32*). Further research would be needed to confirm those findings.

In the community testing data analyzed, testing tended to be conducted shortly after the appearance of symptoms, whereas for surveillance studies, such as the ONS CIS, testing was conducted across the period when a person tested positive on PCR (multiple weeks), leading to a greater range of Ct values. However, with the scale of community testing at the height of the pandemic in England, these data provided earlier indications of new variants than survey data and provided a more useful early indicator of growth of Delta or Omicron than lateral flow test/PCR test positivity, reflex assays, or sequencing. After a new variant was dominant, positivity and case rates became more useful for monitoring. The time delay for detecting a break point by using ISR was ≈2 weeks, but a change could be observed before then. For our analyses, we used gamma distributions, based on the best visual fit, although Poisson or negative binomial distributions may have been more appropriate for the daily count of positive tests. However, given the scale of the data in this analysis, the final fit by model is not likely to differ substantially.

Smaller studies have suggested that changes in Ct values can indicate changes in trajectory of the epidemic, including a study from Spain (*33*) that reported differential changes in mean Ct values and rate of positive tests in the first and second waves; a study from Pakistan (*34*), a study from Italy (*35*), and a study from Belgium (*36*) that reported a 17-day lag between changes in Ct values and positive tests, and a study from the United States that reported a 33-day lag between Ct values and cases (*37*). Our study, which used national surveillance data for >1 year, along with studies from ONS CIS and other studies in the United Kingdom (*4*, *28*) (A.S. Walker AS, unpub. data), provides evidence that using S-specific Ct values can identify emerging new variants and further evidence that Ct values can detect changes in incidence before positive test counts and sequencing data. We found that monitoring COVID-19 gene-specific Ct values in England was a useful early indicator for detecting epidemic growth and providing insight to support public health policy. This useful application of clinical testing data, although of maximal benefit in the context of the first waves of the COVID-19 pandemic, may have other applications for infectious disease monitoring.

Appendix Additional results for study of cycle threshold values as indication of increasing SARS-CoV-2 new variants, England, 2020–2022.
