# Supplementary material for: Cycle Threshold Values as Indication of Increasing SARS-CoV-2 New Variants, England, 2020–2022
Source: Emerg Infect Dis. 2023 Oct;29(10):2024–31. doi: 10.3201/eid2910.230030 (PMC10521603; doi:10.3201/eid2910.230030)
Supplement: Appendix — Additional results for study of cycle threshold values as indication of increasing SARS-CoV-2 new variants, England, 2020–2022. [file 23-0030-Techapp-s1.pdf]

Article DOI: https://doi.org/10.3201/eid2910.230030

EID cannot ensure accessibility for supplementary materials supplied by authors. Readers who have difficulty accessing supplementary content should contact the authors for assistance.

Cycle Threshold Values as Indication of Increasing SARS-CoV-2 New Variants, England, 2020–2022

Appendix

Appendix Table. Rapid genotyping and whole genome sequencing data for (a subset of) tests for SARS Co-V-2 from clinical testing, by calendar period (data from UKHSA)

Variant	Lineage	WHO name	1 Sep 2020–31 Jan 2021		1 Feb 2021–30 Nov 2021		1 Dec 2021–31Jan 2022	
VOC-20DEC-01	B.1.1.7	Alpha	78,410	85.8%	151,968	9.8%	13	0.001%
VOC-21FEB-02	B.1.1.7 with E484K	Alpha with E484K	30	0.03%	21	0.001%		0.0%
VOC-20DEC-02	B.1.351	Beta	191	0.2%	766	0.05%	1	0.0001%
VOC-20DEC-02 or VUI-21JUL-01	B.1.351 or B.1.621	Beta or Mu			12	0.001%	9	0.0005%
VOC-21APR-02	B.1.617.2	Delta	50	0.1%	1,273,965	82.4%	652,565	33.3%
VUI-21OCT-01	AY.4.2	Delta AY 4.2	1	0.001%	65,406	4.2%	84,924	4.3%
VUI-21FEB-03	B.1.525	Eta	58	0.06%	404	0.03%		
VOC-21JAN-02	P.1	Gamma	1	0.001%	254	0.02%	21	0.001%
VUI-21APR-01	B.1.617	Kappa			428	0.03%		
VUI-21JUN-01	C.37	Lambda			8	0.001%		
VUI-21JUL-01	B.1.621	Mu			48	0.003%	2	0.0001%
VOC-21NOV-01	B.1.1.529, BA.1	Omicron	107	0.1%	10,970	0.7%	1,120,807	57.1%
VUI-22JAN-01	BA.2	Omicron	1	0.001%	428	0.03%	12,117	0.6%
VUI-21MAR-02	P.3	Theta			8	0.001%		
VUI-21JAN-01	P.2	Zeta	30	0.03%	19	0.001%		
VUI-21FEB-04	B.1.1.318		7	0.008%	319	0.0%	8	0.0004%
VUI-21MAR-01	B1.324.1 with E484K		2	0.002%				
VUI-21FEB-01	A.23.1 with E484K		73	0.08%	8	0.001%		
VUI-21MAY-01	AV.1				182	0.01%		
VUI-21APR-03	B.1.617.3				16	0.001%		
VUI-21MAY-02	C.36.3				127	0.01%		
Not classified as a variant of concern			90,862	99.4%	40,811	2.6%	91383	4.7%
Total			169,823		1,546,168		1,961,850	

Note: “other” variants in supplementary figure 1 and main text are all variants of concern or under investigation excepting Alpha, Delta and Omicron BA.1.

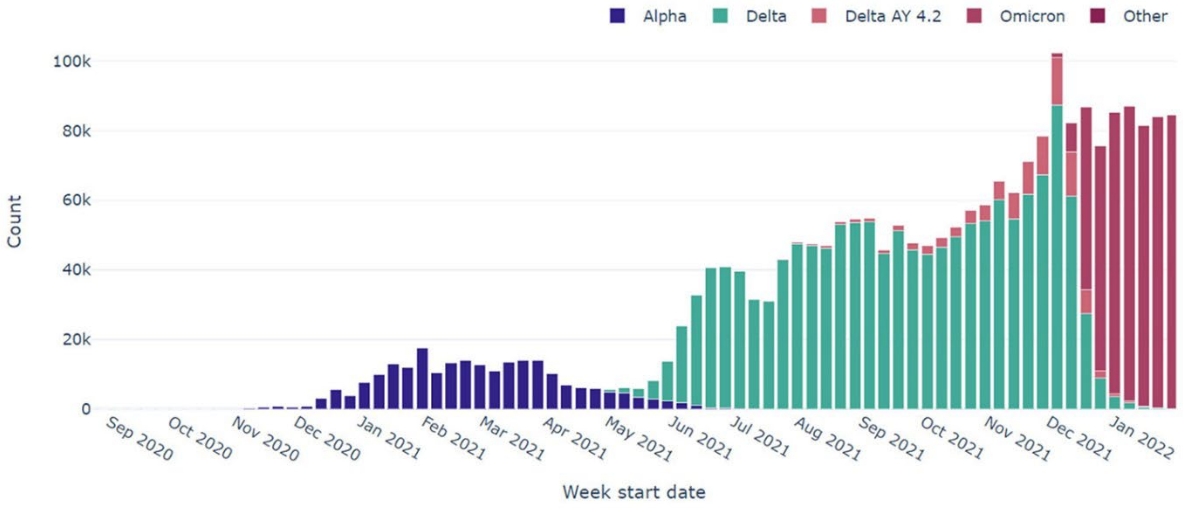

Appendix Figure. Variant data from whole genome sequence (includes full whole-gene sequencing and rapid genotyping) results for SARS CoV-2 tests from England from 31 August 2020 through 31 January 2022. Other includes all other variants and those not classified as variants of concern.
